# Neurocysticercosis with a single brain lesion in Germany: a case report

**DOI:** 10.4076/1757-1626-2-8692

**Published:** 2009-09-09

**Authors:** Felix Luessi, Janina Sollors, Katrin Frauenknecht, Eike Schwandt, Harald D Mueller, Peter Stoeter, Johannes Blum, Frank Thoemke

**Affiliations:** 1 Department of Neurology, Johannes Gutenberg-University Mainz, Langenbeckstr. 1, 55101 Mainz, Germany; 2 Department of Neuropathology, Johannes Gutenberg-University Mainz, Langenbeckstr. 1, 55101 Mainz, Germany; 3 Department of Neurosurgery, Johannes Gutenberg-University Mainz, Langenbeckstr. 1, 55101 Mainz, Germany; 4 Institute of Neuroradiology, Johannes Gutenberg-University Mainz, Langenbeckstr. 1, 55101 Mainz, Germany; 5 Swiss Tropical Institute, Socinstr. 57, 4002 Basel, Switzerland

## Abstract

Neurocysticercosis is rare in Western Europe and a high degree of physician awareness is necessary for diagnosis. We describe a case of Neurocysticercosis with a single brain lesion acquired in Germany in which only surgical removal and subsequent histological examination allowed diagnosis whereas diagnostic investigation yielded no pathological findings.

## Introduction

Neurocysticercosis (NCC) is the most common CNS parasitosis worldwide. It is caused by infection with eggs of the tapeworm Taenia solium, found in undercooked pork, affecting the gut initially and spreading haematogenously [1]. Sufferers often experience a long asymptomatic period, and can present with a variety of neurological manifestations, including focal neurological deficits and seizures. While NCC is the most frequent cause of adult-onset seizures in Latin America, South East Asia and Africa, it is rare in Western Europe and mainly occurs in immigrants from endemic regions [2].

## Case presentation

A 69-year-old German patient presented with a first generalized epileptic seizure. He grew up on a farm with pigs. His travel history revealed no trips to foreign countries.

**Figure 1:**
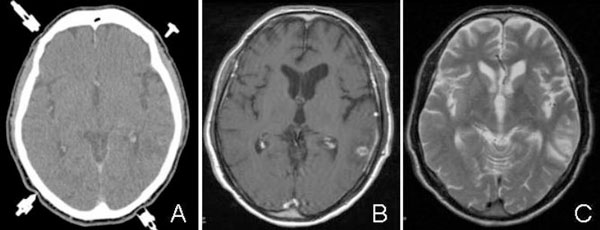
**(A) Contrast-enhanced axial CT scan showing a round lesion in the left temporal lobe**. **(B)** Contrast-enhanced axial T1-weighted MR image shows a sharply defined ring enhancement. **(C)** Axial T2-weighted MR image demonstrates a perifocal edema.

**Figure 2:**
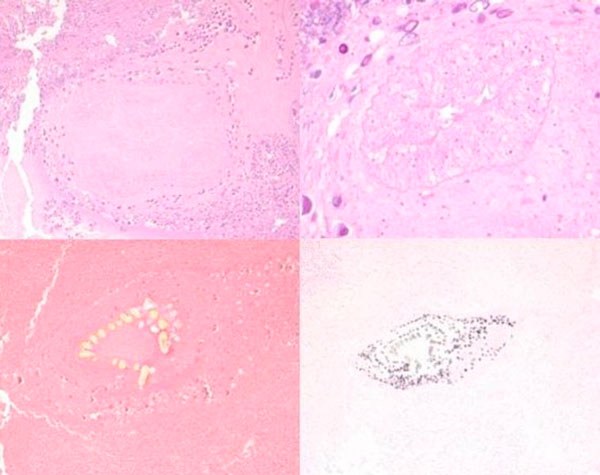
**(A) The scolex of a tapeworm with four suckers is clearly visible, surrounded by granulomatous inflammatory infiltrates, consisting of numerous plasma cells, lymphocytes and in part eosinophilic granulocytes, and tissue debris**. The body of the tapeworm with its segments (proglottids) is not visible, H & E. **(B)** Close-up photograph of one of the four well preserved suckers, H & E. **(C)** One row of hooks on the scolex is detectable, EvG. **(D)** Some calcifications are found in the vicinity of the head of the tapeworm, Kossa.

On examination the patient was afebrile, fully conscious, orientated and showed no neurological abnormalities. MRI disclosed a solitary cystic lesion with gadolinium enhancement in the left temporal lobe surrounded by a perifocal edema (Figure 1). EEG showed intermittent left temporal slowing without epileptic activity. Hematologic and blood chemical tests, a lumbar puncture and stool sample analyses gave no pathological results. Despite extensive microbiological examinations no infectious agents could be detected. Both chest radiography and abdominal sonography were normal. Before surgery a CT scan stereotactically localized the lesion, which was subsequently removed with sonography-assisted microsurgical techniques via temporal osteoclastic craniotomy. Intraoperatively a solid, round shaped lesion with a light-yellow colored, glossy surface was observed. Histological examination revealed a scolex of a pork tapeworm surrounded by inflammatory chronic granulomatous infiltrates (Figure 2). Based on the identification of a larval stage of Taenia solium in biopsy material NCC was diagnosed. An enzyme-linked immunoelectrotransfer blot (EITB) assay did not detect specific antibodies against Taenia solium in serum and CSF. Chest and abdominal CT and radiography of the legs revealed no extraneural involvement.

The patient received an antiparasitic treatment with albendazole 800 mg/d in combination with dexamethasone 4 mg every 8 h for 4 weeks and remained seizure-free under anticonvulsant therapy with lamotrigine 100 mg/d.

## Discussion

The differential diagnosis of a solitary cystic cerebral lesion on CT or MRI includes abscess, tubercle, metastasis and glioblastoma. Parasitic CNS infections and subacute cerebrovascular events should also be considered. The clinical picture of NCC is variable with seizures, focal neurological signs, and intracranial hypertension depending on the amount and localization of the cysts [2]. According to post mortem studies, 80% of neurocysticercal infections remain asymptomatic [3]. Human cysticercosis occurs either via endogenous or exogenous autoinfection in tapeworm carriers or by ingesting Taenia solium eggs after fecal oral transmission. Diagnosis of NCC is often based on the clinical presentation, neuroimaging abnormalities and serology [1]. Serological techniques can vary depending on the activity of the cyst and the number of lesions [4]. In a study of patients with histologically confirmed NCC, 94% with two or more lesions had specific antibodies detectable by EITB compared to only 28% with a single lesion [5]. Thus, negative results on serological testing do not rule out NCC and sometimes, as in our case, more invasive procedures, such as surgical removal or stereotactic brain biopsy, are required to confirm the diagnosis. Specific anthelminthic therapy with albendazole or praziquantel is recommended for patients with non-calcified, viable cystic lesions [6,7]. Accompanied corticosteroids prevent increased inflammation due to cyst degeneration under anthelminthic treatment [8]. Surgical intervention can be necessary in the setting of intracranial hypertension caused by hydrocephalus or giant cysts [9]. Although the diagnosis of NCC is rare in Western Europe and mainly occurs in travelers and immigrants from endemic regions, the disease should even be considered in the differential diagnosis of adult-onset seizures with a single cystic brain lesion in patients without travel history [10,11].

## Abbreviations

CNS: central nervous system; CSF: cerebrospinal fluid; CT: computed tomography; EEG: electroencephalography; EITB: enzyme-linked immunoelectrotransfer blot; MRI: magnetic resonance imaging; NCC: neurocysticercosis.

## Consent

Written informed consent was obtained from the patient for publication of this case report and accompanying images. A copy of the written consent is available for review by the Editor-in-Chief of this journal.

## Competing interests

The authors declare that they have no competing interests.

## Authors' contributions

FL, JS, ES, JB and FT were major contributors in writing the manuscript. KF and HM performed the histological examination of the brain biopsy. PS performed the analysis of CT and MRT scans. All authors read and approved the final manuscript.
